# Arterial Spin Labeling and Dynamic Susceptibility Contrast-enhanced MR Imaging for evaluation of arteriovenous shunting and tumor hypoxia in glioblastoma

**DOI:** 10.1038/s41598-019-45312-x

**Published:** 2019-06-19

**Authors:** S. Ali Nabavizadeh, Hamed Akbari, Jeffrey B. Ware, MacLean Nasrallah, Samantha Guiry, Stephen J. Bagley, Arati Desai, Scott Levy, Whitney Sarchiapone, Timothy Prior, John Detre, Ronald L. Wolf, Donald M. O’Rourke, Steven Brem, Christos Davatzikos

**Affiliations:** 1 Department of Radiology, Hospital of University of Pennsylvania, Perelman School of Medicine of the University of Pennsylvania, Philadelphia, Pennsylvania USA; 2 Division of Neuropathology, Department of Pathology and Laboratory Medicine, Perelman School of Medicine, University of Pennsylvania, Philadelphia, PA 19104 USA; 3 Abramson Cancer Center, University of Pennsylvania, Philadelphia, PA 19104 USA; 4 Department of Neurology, Hospital of University of Pennsylvania, Perelman School of Medicine of the University of Pennsylvania, Philadelphia, Pennsylvania USA; 5 Department of Neurosurgery, Hospital of University of Pennsylvania, Perelman School of Medicine of the University of Pennsylvania, Philadelphia, Pennsylvania USA

**Keywords:** CNS cancer, Cancer imaging

## Abstract

Glioblastoma (GBM) is the most common primary malignant brain tumor in adults and carries a dismal prognosis. Significant challenges in the care of patients with GBM include marked vascular heterogeneity and arteriovenous (AV) shunting, which results in tumor hypoxia and inadequate delivery of systemic treatments to reach tumor cells. In this study, we investigated the utility of different MR perfusion techniques to detect and quantify arteriovenous (AV) shunting and tumor hypoxia in patients with GBM. Macrovascular shunting was present in 33% of subjects, with the degree of shunting ranging from (37–60%) using arterial spin labeling perfusion. Among the dynamic susceptibility contrast-enhanced perfusion curve features, there were a strong negative correlation between hypoxia score, DSC perfusion curve recovery slope (r = −0.72, P = 0.018) and angle (r = −0.73, P = 0.015). The results of this study support the possibility of using arterial spin labeling and pattern analysis of dynamic susceptibility contrast-enhanced MR Imaging for evaluation of arteriovenous shunting and tumor hypoxia in glioblastoma.

## Introduction

Glioblastoma (GBM) contains irregular vascular proliferations that are not able to sufficiently oxygenate rapidly growing tumor tissue, resulting in a positive feedback loop of hypoxia and aberrant angiogenesis. An additional component of abnormal vasculature in GBM is arteriovenous (AV) shunting, which has been known for decades by the presence of early venous drainage in cerebral angiogram and the intraoperative observation of “red veins”^1^. The presence of AV shunting in patients with GBM may have important therapeutic implications. First of all, in the presence of AV shunting, a significant amount of intra-arterially or systemically administered chemotherapeutic/antiangiogenic agents will not perfuse tumor cells and instead, will enter the systemic circulation through the aberrant AV channels. Secondly, AV shunting leads to increased risk during surgical resections^2^, which must be performed meticulously in order to control bleeding in the presence of arterialized veins, especially when close to eloquent brain regions. Finally, arteriovenous shunting results in poor delivery of oxygen to the tumor and can be associated with tumor hypoxia in GBM^3^. It has been shown that the level of hypoxia varies, and only a subset of patients with GBM demonstrates severe tumor hypoxia^4^. Severe hypoxia in GBM is associated with aggressiveness^4^, induces resistance to radiotherapy^5^, and is related to poor prognosis^6,7^. Given the importance of hypoxia in tumor behavior, development of reliable and noninvasive imaging techniques to evaluate tumor hypoxia is crucial.

Previous case series using selective intra-arterial, intratumoral injection of 99mTc-labeled microparticles (macroaggregated albumin) have revealed significant AV shunting ranging from 47–89% in patients with GBM^1,8^. More recently, arterial spin Labeling (ASL) perfusion and susceptibility weighted imaging (SWI) have been successfully applied to detect and quantify AV shunting in arteriovenous malformations and arteriovenous fistulas in the brain by multiple investigators, including our group^9–12^. In addition, dimensionality reduction methods using dynamic susceptibility contrast-enhanced (DSC) MRI were applied to analyze perfusion in patients with GBM to identify tissue features such as peritumoral tissue heterogeneity and locations of future recurrence^13,14^. In this study, we investigated the utility of ASL perfusion and principal component analysis (PCA) of DSC perfusion images to detect and quantify macrovascular AV shunting, as well as to infer capillary-level hemodynamics at the voxel level in patients with glioblastoma.

## Results

Of the 16 patients who underwent MRI imaging, one patient was excluded due to motion degraded ASL images, one patient declined surgery given the extensive multifocal nature of the tumor, and two patients were excluded because the histopathology demonstrated diffuse midline glioma. Among the 12 included patients, seven were male and five were female (mean age = 63.25 ± 12.1, median age = 62 years).

**Figure 1:**
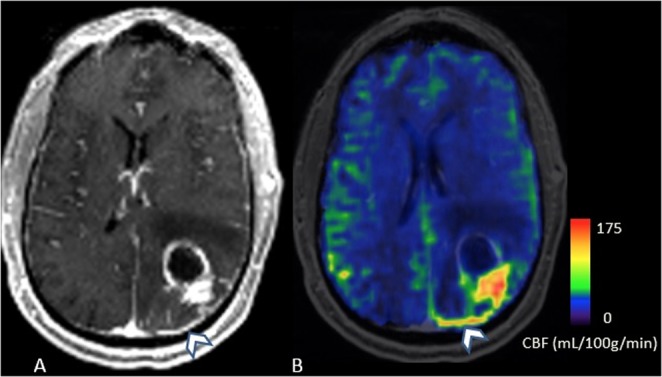
Left parietal GBM in 55-year-old subject. Post-contrast T1 MPRAGE demonstrates a left parietal tumor with a prominent vein draining the posterior aspect of the tumor, which is bright on CBF map (**A**,**B**, arrowhead). The shunt fraction was 37% (**B**).

**Figure 2:**
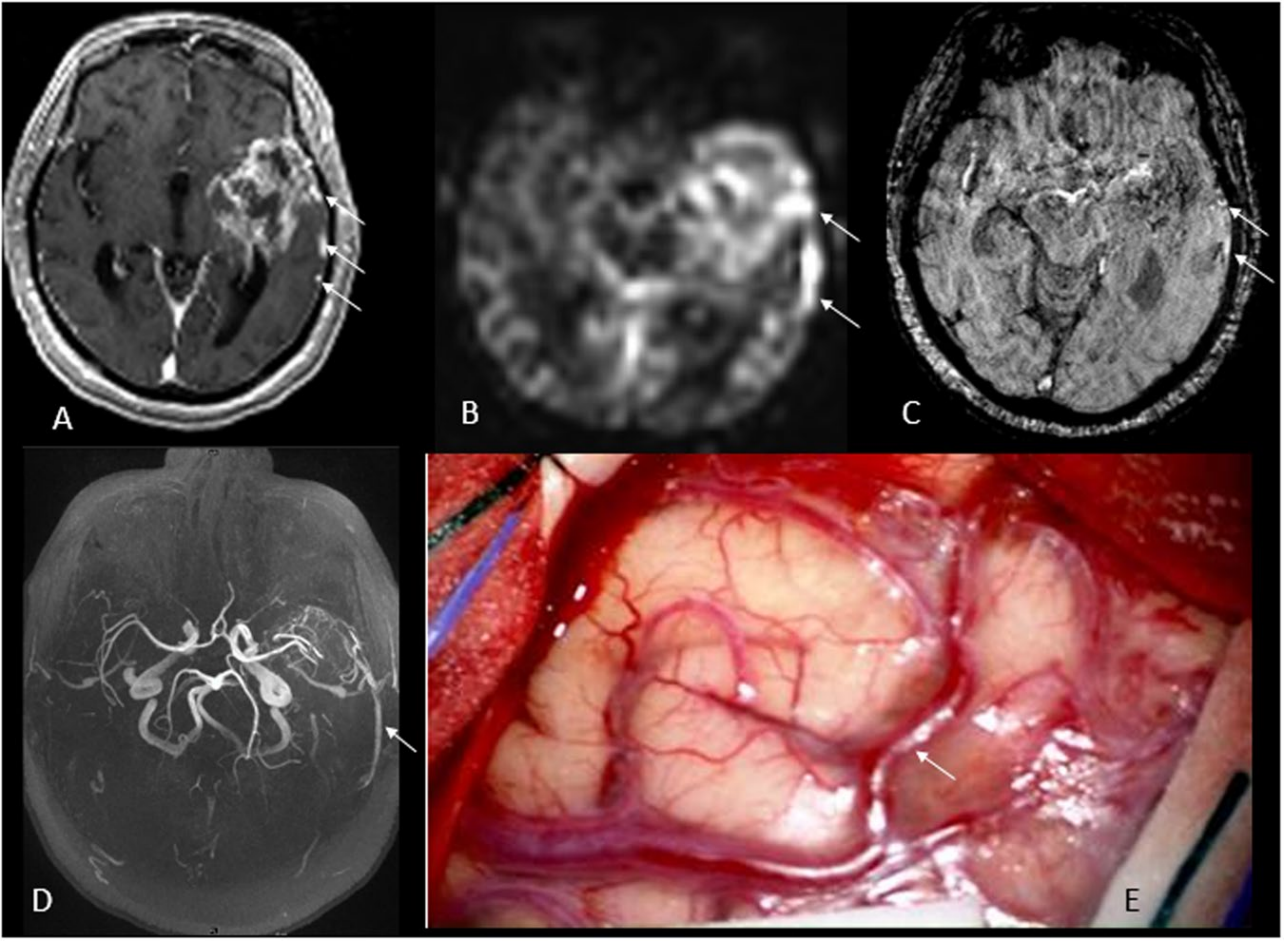
Left temporal lobe GBM in a 60-year-old subject. Post-contrast T1 MPRAGE demonstrates a heterogeneously enhancing mass in left temporal lobe with multiple prominent veins laterally, which are very bright on ASL and SWI images (**B**,**C**, arrows). 3D TOF MRA demonstrates prominent veins lateral to the tumor which drain into the vein of Labbé (**D**, arrow). Intraoperative image displays cortical surface with arterialized vein of Labbé (**E**, arrow) in the central part of the field and telangiectactic vessels in the upper right.

Macrovascular shunting was present in 4/12 (33%) subjects, with degree of shunting ranging from 37–60% (Figs 1 and 2). The average hypoxia score was higher in patients with macrovascular shunting compared to subjects without shunting (49.25 vs 40.1), but the difference was not statistically significant (P > 0.05). Among the DSC perfusion curve features, there was a strong negative correlation between hypoxia score and DSC perfusion curve recovery slope (r = −0.72, P = 0.018), as well as angle (−0.73, P = 0.015). In addition, the skewness of recovery slope was positively correlated with hypoxia score (r = 0.70, P = 0.023). Among other histopathologic features, Ki-67 was positively correlated with the mean angle (r = 0.71, P = 0.028) and slope (r = 0.82, P = 0.006) of the DSC perfusion curve recovery.

Voxel-based analysis of enhancing tumor demonstrated significant difference between the angle and slope of DSC curve recovery in patients with macrovascular shunting compared to the patients without shunting (P < 0.001), Table 1).

**Table 1:** Voxel-based analysis of enhancing tumor in patients with macrovascular shunting compared to the patients without shunting.

	Shunting			Non-shunting			P value
	Mean ± SD	Median	Skewness	Mean ± SD	Median	Skewness	
Angle (degree)	79.25 ± 12.47	82.80	−4.52	80.03 ± 13.02	83.57	−4.65	<0.001
Slope	8.72 ± 5.29	7.91	1.19	10.06 ± 6.33	8.88	1.09	<0.001

**Figure 3:**
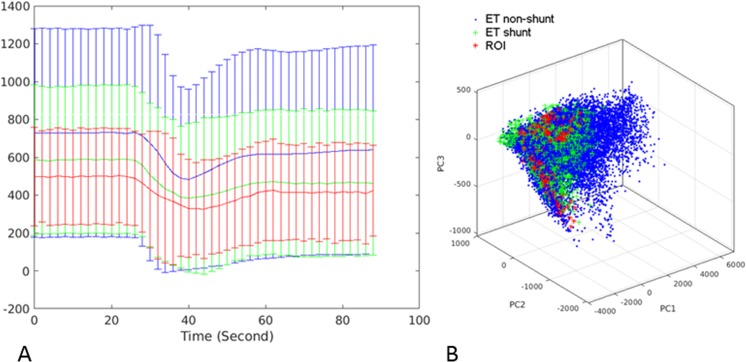
Perfusion Time-Series and Calculated Principle Components. The left panel (A) shows the average perfusion signal of all voxels for a given region of interest with two standard deviations bars. Enhancing tumor voxels of non-shunted patients (ET non-shunt) are shown in blue, enhancing tumor voxels of shunted patients (ET shunt) are shown in green, and ASL based ROI of the arterialized veins of shunted patients were shown in red. The right panel (B) displays the calculated principal components for each tissue type, based on the perfusion signal. PC1, PC2, and PC3 denote the first, second, and third principal components, respectively. ET nonshunt: Enhancing voxels of non-shunted patients. ET shunt: Enhancing voxels of shunted patients. ROI: ASL based ROI on the arterialized veins of shunted patients.

The right panel of Fig. 3 shows the principal components (PC) based on the perfusion signal time-curve of all subjects’ enhancing tumor and shunting voxels. The skewness of the third principal component was significantly more negative in patients without shunting (−0.65 vs −0.085, P = 0.006) and was positively correlated with microvascular proliferation in histopathologic analysis (r = 0.67, P = 0.031).

**Figure 4:**
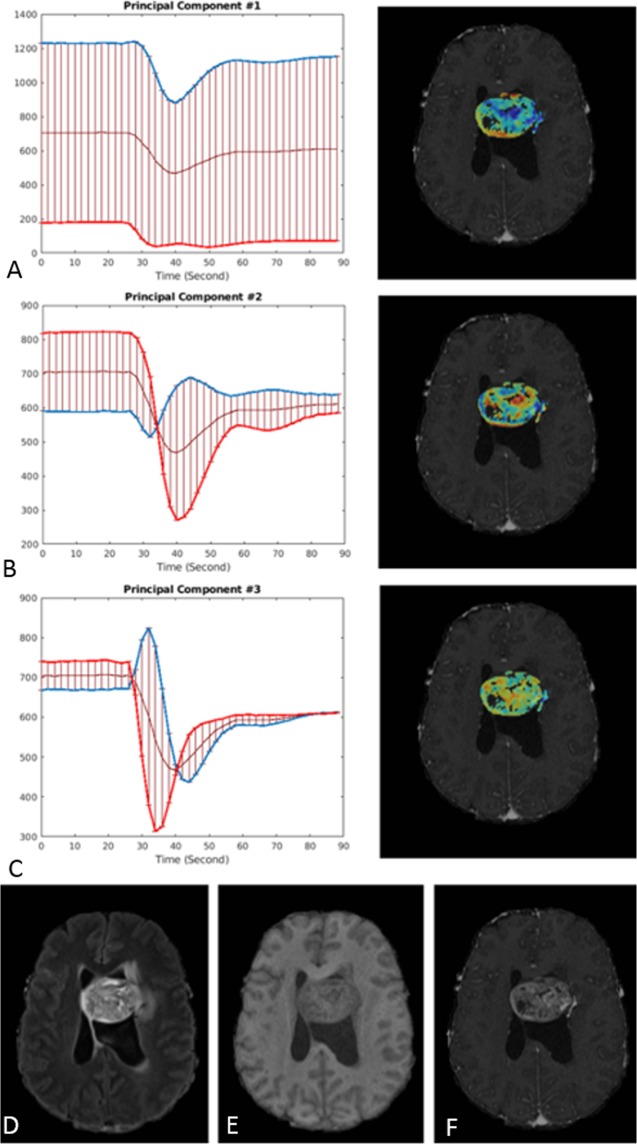
Principal component images and plots that demonstrate the first (**A**), second (**B**) and third (**C**) principal components overlaid on the T1CE MR image (right), along with the plot of the corresponding principal eigenvector (left), are shown to illustrate the breadth of information contained within the perfusion time-series. The plots have been constructed from the perfusion signal of all voxels, with the error bar representing +/− two standard deviations of the respective principal component. Red and blue lines represent the negative and positive parts of PCs respectively. The bottom panel shows FLAIR (**D**), T1 (**E**), and T1 post contrast images (**F**).

In Fig. 4, the mean of perfusion signals and first three principal components obtained from all voxels multiplied by +/− two standard deviations of the respective principal components are plotted. Principal component 1 was found to relate primarily to the global baseline signal level at each voxel. Principal component 2 has the greatest variability around the baseline and the depth of the curve; therefore, it conveys the depth of the signal decrease in relation to the baseline level, while the third principal component reflects the shape of the perfusion signal and the steepness of the signal recovery to a greater degree.

## Discussion

In this study, we showed that ASL can detect macrovascular shunting in a subset of GBM patients, and that tumors with and without shunting have different DSC perfusion curve characteristics. In addition, we also showed that the slope and angle of the DSC perfusion curve are strongly correlated with tumor hypoxia and Ki-67 proliferation index. Although the hypoxia score was higher in patients with macrovascular shunting; however, the difference was not statistically significant, which could be due to lack of adequate statistical power in our study.

Two previous small studies used direct injection of 99mTc-labeled microparticles to quantify AV shunting in gliomas based on the notion that the injected microparticles should be trapped in a normal capillary bed but escape through abnormal arteriovenous channels in the presence of AV shunting, eventually becoming trapped and imaged in the lungs. Mariani *et al*. included two patients with glioma, and both demonstrated AV shunting ranging from 63% to 70%^8^. Another study by the same group included seven patients with high grade glioma, which all demonstrated AV shunting ranging from 47–89%^1^. Yoshikawa *et al*. studied 26 patients with GBM using cerebral angiography and used early venous filling as an indicator of AV shunting. They found that 53% of GBMs had AV shunting, and that AV shunting is significantly more common in presylvian tumors compared to other locations^2^. The results of our study are more concordant with those of Yoshikawa *et al*., as we also demonstrated AV shunting in a subset of patients with GBM. This may be due to differences in technique as the nuclear medicine studies described used particles with diameters of 25 to 50 mm, while ASL imaging uses labelled water as a tracer. In addition, our criterion to determine AV shunting was visualization of bright veins, which is an equivalent to early venous filling on cerebral angiography. Improving the spatial resolution and signal to noise ratio of ASL by applying different acquisition techniques such as continuous arterial spin labeling (CASL) and imaging at higher field magnets^15^ may be helpful to improve the detection of smaller veins that carry shunted blood. In addition, application of techniques such as velocity selective ASL^16^ may have an added value by using velocity cut-offs that can be adjusted based on the degree of tumor vascularity. The strong but reverse correlations between the slope and angle of the DSC perfusion curve with tumor hypoxia and Ki-67 proliferation index in our study is consistent with two prior studies that demonstrated low proliferative activity in hypoxic tumors areas compared to well-oxygenated tumor compartments^17,18^.

The proposed PCA data-driven method uses temporal dynamics of DSC MR imaging to extract information relevant to tumor hypoxia not typically obtained with traditional cerebral blood volume (CBV) maps^14^. Thus, our results suggest that more comprehensive analysis of the DSC perfusion curve can provide additional clinically-useful information about the GBM tumor microenvironment and the PCA extracts information may reflect tumor AV shunting, as evidenced by the close relationship in PCA space with the shunted vessels on ASL (Fig. 3). Our study lends a number of implications for clinical practice. The method described can serve as a tool to compare the efficacy of systemic forms of treatment in patients with glioblastoma with different degrees of shunting. In addition, local forms of treatment may be more suitable for patients with high levels of AV shunting rather than systemic treatments^19^. Finally, to determine the potential effect of AV shunting on drug delivery, quantitative pharmacology studies can be performed to determine the biodistribution of drugs under different degrees of AV shunting. For example, temozolamide, currently the standard of care for all newly diagnosed GBM^20^, can be labelled with experimental PET radiotracers^21^ and be used to determine the biodistribution of the drug under different degrees of AV shunting.

The current work has several limitations. The main limitation is the small sample size, noting that this study was prospective and required preoperative consent to perform MR sequences that are not part of the routine brain tumor imaging at our institution, as well as additional histopathology examinations. Another limitation in the widespread use of our approach is the specialized nature of principal component analysis; however, the software package Cancer Imaging Phenomics Toolkit (CaPTk) that is used in this study is freely available for use^22^.

## Conclusion

ASL imaging can be used to determine and quantify macrovascular arteriovenous shunting in patients with GBM. The combination of ASL imaging and DSC perfusion curve analysis might provide important information about the tumor microenvironment and tumor hypoxia. This study is limited by small patient numbers and its preliminary nature, but hints to the possibility of a combined approach using DSC and ASL to make noninvasive predictions of arteriovenous shunting and tumor hypoxia. This clinical significance of this approach in the context of systemic therapies need to be investigated in future studies.

## Methods

Institutional review board of the University of Pennsylvania approval was obtained for this prospective study and all research was performed in accordance with relevant guidelines and regulations. Informed consent was obtained from the participants. 16 patients with intra-axial brain mass suggestive of high grade glioma were referred to the radiology department of Hospital of University of Pennsylvania from Dec 2016 to May 2018 and underwent brain MRI at 3-Tesla (Magnetom TrioTim; Siemens, Erlangen, Germany) using a 12-channel phased array head coil. Routine sequences were obtained, including pre and post-contrast axial T1-weighted 3D MPRAGE (TR/TE/TI = 1760/3.1/950 ms, 192 × 256 matrix size, 1-mm section thickness) and post contrast axial FLAIR (TR/TE/TI = 9420/141/2500 ms, 3-mm section thickness). SWI scanning parameters were (TR) = 27 ms, (TE) = 20 ms, field of view (FOV) = 220 mm, imaging matrix = 256 × 192, slice thickness = 1.5 mm, and GRAPPA factor = 2. Pseudocontinuous ASL (PCASL) was performed using a labeling time = 1.5 s, PLD = 1.5 s, and labeling plane offset = 8 cm, TR/TE = 9181 ms/11 ms, FOV = 220 × 220 mm^2^, matrix size = 64 × 64, voxel size = 3.4 × 3.4 × 5 mm^3^ and 3D spiral read out. For DSC imaging, a bolus of gadobenate dimeglumine (MultiHance; Bracco Diagnostics, Princeton, New Jersey) was injected with a preloading dose of 0.07 mmol/kg, to reduce the effect of contrast agent leakage on CBV measurements^23^. DSC imaging was performed by using a gradient-echo echo-planar imaging sequence during a second 0.07-mmol/kg bolus of contrast agent with the following parameters: TR/TE = 2000/45 ms, FOV = 22 × 22 cm2, resolution = 1.72 × 1.72 × 3 mm^2^, 20 sections.

### Image analysis

#### Determination and quantification of macrovascular shunting

Two neuroradiologists with 7 (SAN) and 3 (JW) years of experience evaluated the MRI images by consensus. Macrovascular AV shunting was defined as the presence of hyperintensity (equivalent to or brighter than contralateral cortex) within the tumor venous drainage on ASL CBF (cerebral blood flow) maps, confirmed by hyperintensity on SWI images (Figs 1 and 2). Normal veins appear dark on SWI images due to high deoxy-hemoglobin content, while arterialized vein appear bright^11^. ASL images were linearly co-registered with the T1 post-contrast image using an affine registration tool FLIRT^24^, available in FSL^25^. Hyperintense draining veins identified on ASL images and confirmed on T1 post-contrast images were segmented in a semi-automated fashion using ITK-SNAP^26^. The enhancing component of the tumor was also segmented in semi-automated fashion. Macrovascular AV shunting was quantified by normalizing the blood flow (BF) in the draining vein to the volume and BF of the tumor and draining vein. The AV shunt fraction was quantified with the following formula: (Vol_vein_ × mean BF_vein_)/(Vol_tumor_ × mean BF_tumor_) + (Vol_vein_ × mean BF_vein_).

#### Analysis of DSC perfusion curve

DSC data was linearly co-registered with the T1 post-contrast image using FLIRT, and was used to extract at each voxel the time to maximum drop of the perfusion signal (T_D_), the time to 95% recovery of the perfusion signal (T_R_), and the respective signal intensities (I_D_, I_R_). The slope of the recovery curve was calculated by slope = (I_R_ - I_D_)/(T_R_ - T_D_), and recovery angle calculated by Angle = ArcTan(slope). Subsequently, the mean, median, standard deviation, and skewness were calculated for slope of the recovery curve, and recovery angle.

#### Calculation of Principal Components

Principal component analysis (PCA) is a dimensionality reduction method that has been used^13,14^ to distill the DSC-MRI time series down to a few principal components that capture the temporal dynamics of blood perfusion. In order to characterize the perfusion characteristics of enhancing tumor (ET) in shunted and non-shunted patients, we also used the ASL-based ROIs as described before. All voxels of ET of patients with and without macrovascular shunting as well as voxels from the tumor venous drainage that demonstrated shunting were utilized to calculate the first three principal components. We performed PCA using these ROIs and then segmented ET of all subjects^13,14^. Cancer Imaging Phenomics Toolkit (CaPTk) software was used for initial calculations of PC analysis^27^.

#### Evaluation of Principal Components

The principal components incorporate and convey complex, high-dimensional information from various aspects of the dynamics perfusion time-series, such as baseline signal, depth of signal decrease, slope, and angle of signal recovery. The first principal components were plotted in Fig. 3 (right panel), which displays ET of shunted patients (green) and non-shunted patients (blue), as well as the ROI of tumor venous drainage with shunting (red). The left panel of Fig. 3 shows the mean perfusion curve with two standard deviations bars for the same groups. PCA was subsequently used to capture the information of the perfusion time series in all subjects. Because of the relative consistency in the perfusion pattern of the various regions, a feature vector consisting of first three principal components was sufficient to capture more than 98% of the variance in the perfusion signal for all tissue types and all patients (Fig. 3, Right panel, depicts the first three components).

#### Histopathological analysis

**Figure 5:**
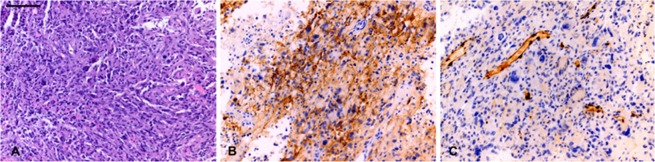
Immunohistological assessment of tumor and hypoxia was performed on hematoxylin and eosin stained sections of tumor (**A**), CAIX immunostaining for hypoxia (**B**), and ERG immunostaining highlighting vessels (**C**). Scale bar: 100 microns, upper left.

All surgical specimens were reviewed by a neuropathologist (M.P.N) who was blinded to the shunting data. The tissue was stained with hematoxylin and eosin, as well as with the following antibodies: K-67 (Dako IR62661, clone MIB-1, prediluted), ERG (BIOCARE, PM421AA, prediluted), CAIX (Leica, CAIX-L-CE, 1:100). Unstained slides were pretreated with heat retrieval, epitope retrieval 1, in citrate buffer pH 6.0 (Leica microsystems) for 20 min. Immunohistochemical staining was performed on the Bond 111 Autostainer with the DAB chromogen and a hematoxylin counterstain. The overall tumor Ki-67 proliferation index was semi-quantitatively estimated based on inspection of a stained section from one tumor block. ERG staining for endothelial cells was performed in order to assess vascular density in dense tumor (Fig. 5). Small, medium, and large caliber vessels in the tumor were each scored on a range of 0–6, with 0 referring to rare vessels present, and 6 referring to dense vascularity. Staining for CA IX was performed to evaluate hypoxia. CAIX catalyzes the reversible hydration of carbon dioxide and is considered to be a reliable cellular biomarker of tumor hypoxia^28^. Expression of CAIX was determined by semi-quantitatively assessing the percentage of stained tumor cells and their staining intensity with an H-score ranging from 0–300. The percentages of weak (1+), moderate (2+), and strong (3+) staining were assessed, and the H-score was calculated as: (weak %)*1 + (moderate %)*2 + (strong %)*3.

### Statistical analysis

Voxel-based statistical analyses were performed through the Mann–Whitney U test to investigate the difference of T_D_ slopes and angles in shunted and non-shunted patients. We also calculated Pearson pairwise linear correlation coefficients between each pair of imaging features and pathology characteristics. Matlab 2018 (The MathWorks, Natick, MA, USA) was used to perform statistical analysis.
